# Long‐Term Ocular Outcomes of Dupilumab in Atopic Dermatitis: Results From the BioDay Registry

**DOI:** 10.1002/clt2.70208

**Published:** 2026-09-29

**Authors:** Nienke Veldhuis, Roselie E. Achten, Chantal M. van Luijk, Marlot M. van der Wal, Constance F. den Hartog Jager, Nicolaas P. A. Zuithoff, Mohsin El Amrani, Edward F. Knol, Femke van Wijk, Marlies de Graaf, Inge M. Haeck, Mor M. Dickman, Marjolein S. de Bruin‐Weller

**Affiliations:** ^1^ Department of Dermatology and Allergology National Expertise Center for Atopic Dermatitis University Medical Center Utrecht Utrecht the Netherlands; ^2^ Department of Ophthalmology University Medical Center Utrecht Utrecht the Netherlands; ^3^ Center for Translational Immunology University Medical Center Utrecht Utrecht University Utrecht the Netherlands; ^4^ Julius Center for Health Sciences and Primary Care University Medical Center Utrecht Utrecht the Netherlands; ^5^ Division Laboratories, Pharmacy and Biomedical Genetics Department of Clinical Pharmacy University Medical Center Utrecht Utrecht University Utrecht the Netherlands; ^6^ Department of Dermatology and Allergology Wilhelmina Children's Hospital University Medical Center Utrecht Utrecht University Utrecht the Netherlands

**Keywords:** adults, atopic dermatitis, conjunctivitis, dupilumab, ocular surface disease

## Abstract

**Background:**

Dupilumab‐associated ocular surface disease (DAOSD) is the most reported side effect in dupilumab‐treated atopic dermatitis (AD) patients. We investigated the effect of long‐term dupilumab treatment on ocular surface disease (OSD), conjunctival goblet cells (GCs), and tear fluid dupilumab levels and proteins.

**Methods:**

This prospective study included moderate‐to‐severe AD patients at the University Medical Center Utrecht, the Netherlands. Patients initiating dupilumab between February 2020–October 2022 were eligible to participate in scheduled ophthalmological examinations alongside routine dermatological examinations at baseline, week 28, and last follow‐up (≥ 2 years). OSD severity was assessed using the Utrecht Ophthalmic Inflammatory and Allergic disease (UTOPIA) score, with DAOSD defined as a ≥ 3‐point increase from baseline. Conjunctival GC quantity and function were assessed with impression cytology. Dupilumab levels and proteins were measured in tear fluid.

**Results:**

Forty‐four patients were included. OSD was present in 90.9% (*n* = 40/44) of patients at all visits. DAOSD occurred in 22.7% (*n* = 10/44) of patients; 40.0% (*n* = 4/10) persisted or newly developed at last follow‐up. Tear break‐up time decreased while tear production increased. Tear fluid dupilumab levels and AD‐related inflammatory proteins decreased over time. Conjunctival GCs decreased at last follow‐up compared to baseline. Mucin5AC (MUC5AC) production in CK19‐CD45‐MUC5AC + cells decreased at week 28 and remained stable at last follow‐up.

**Conclusion:**

OSD remained common during long‐term dupilumab treatment in AD patients. Some patients developed worsening of OSD compatible with DAOSD, including delayed onset, highlighting the need for continued awareness of ocular side effects among clinicians. Reduced MUC5AC production and declining AD‐related inflammatory proteins in tear fluid suggest changes in conjunctival homeostasis and ocular inflammation.

## Graphical abstract

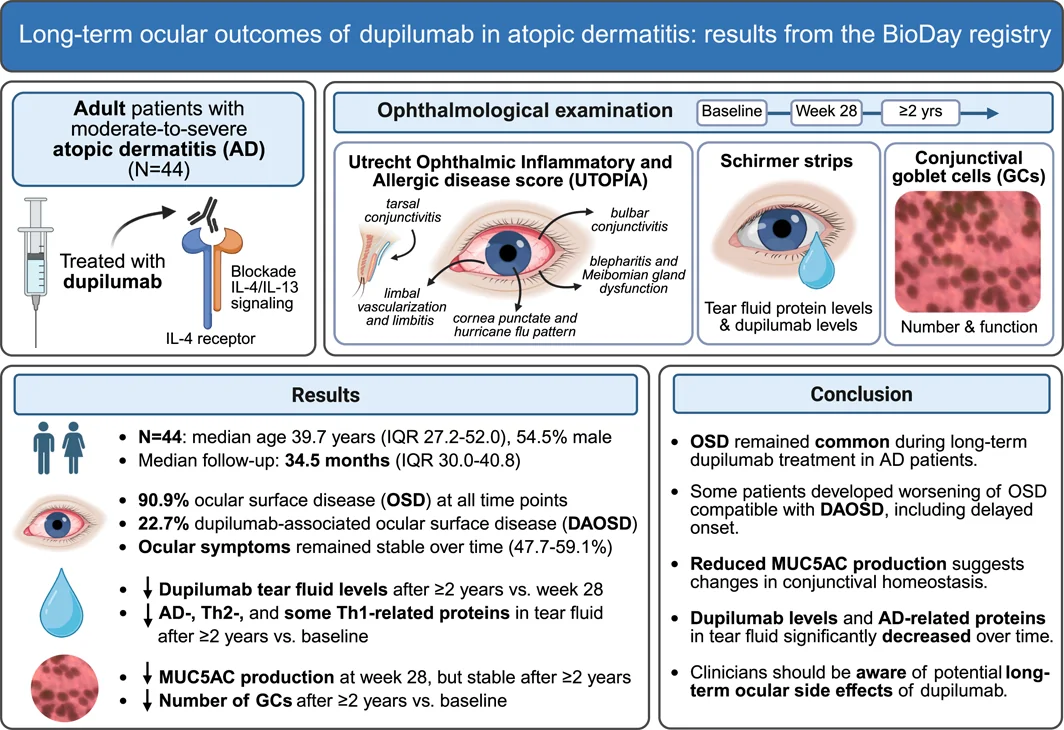

**Abbreviations**
AD: Atopic dermatitis
CI: Confidence interval
CIC: Conjunctival impression cytology
CK19: Cytokeratin 19
CXCL10: C‐X‐C motif chemokine ligand 10
DAOSD: Dupilumab‐associated ocular surface disease
EASI: Eczema Area and Severity Index
GC: Goblet cell
GranB: Granzyme B
HC: Healthy control
IGA: Investigator's Global Assessment
IL: Interleukin
IQR: Interquartile range
METC: Medical Research Ethics Committee
MFI: Median fluorescence intensity
MUC5AC: Mucin 5AC
OSD: Ocular surface disease
PARC: Pulmonary and activation‐regulated chemokine
Q2W: Every 2 weeks
Q3W: Every 3 weeks
Q4W: Every 4 weeks
SD: Standard deviation
TARC: Thymus and activation‐regulated chemokine
TBUT: Tear break‐up time
Th: T helper
UMCU: University Medical Center Utrecht
UTOPIA: Utrecht Ophthalmic Inflammatory and Allergic disease

## 1 Introduction

Atopic dermatitis (AD) is a common chronic inflammatory skin disorder that affects up to 10% of adults [1]. In recent years, new targeted therapies have become available for moderate‐to‐severe AD patients, including dupilumab treatment [2]. Dupilumab inhibits interleukin (IL)‐4 and IL‐13 signaling by blocking the IL‐4 receptor‐alpha. Despite its clinical effectiveness, ocular side effects have been frequently reported in AD patients treated with dupilumab, with incidence rates up to 42% [3, 4, 5, 6, 7, 8, 9].

The exact pathomechanism of dupilumab‐associated ocular surface disease (DAOSD) remains unclear, but it is hypothesized that dupilumab affects conjunctival goblet cells (GCs). GCs produce Mucin 5AC (MUC5AC), which is essential for protecting the ocular surface and maintaining tear film stability [10]. Interestingly, 90% of moderate‐to‐severe AD patients already showed clinical characteristics of ocular surface disease (OSD) before starting dupilumab treatment, which was accompanied by a low number of conjunctival GCs compared to healthy controls (HCs) [11]. After 28 weeks of dupilumab treatment, these conjunctival GC numbers remained stable but low, with a decrease in the percentage of MUC5AC‐producing cells. These findings suggest that an impaired GC function may contribute to the development of DAOSD [6].

Little is known about the course of DAOSD in AD patients undergoing long‐term dupilumab treatment. Beck et al. reported remission of DAOSD in up to 87% of patients after 5 years of dupilumab treatment [12, 13]. However, this finding was based on patient‐reported outcomes rather than objective ophthalmological examinations, and patients with a history of severe DAOSD or treatment discontinuation due to DAOSD were excluded. In contrast, Achten et al. examined 33 dupilumab‐treated AD patients and found that most ophthalmologically confirmed DAOSD cases persisted after 18 months of follow‐up, despite ophthalmic treatment [14]. Overall, prospective studies investigating the long‐term ocular effects of dupilumab based on ophthalmological examinations are lacking.

The chronic and recurrent character of moderate‐to‐severe AD often requires prolonged systemic treatment. This highlights the need for a better understanding of the long‐term ocular safety of dupilumab. Therefore, we aimed to evaluate the frequency and severity of DAOSD in moderate‐to‐severe AD patients after at least 2 years of dupilumab treatment. Additionally, we investigated the long‐term effects on conjunctival GC density and function, as well as tear fluid proteins and dupilumab levels.

## 2 Methods

### 2.1 Study Design and Patients

This prospective, observational cohort study included adult patients (≥ 18 years) with moderate‐to‐severe AD from the BioDay registry treated with dupilumab at the Dermatology department of the University Medical Center Utrecht (UMCU), the Netherlands. All patients who initiated dupilumab between February 2020 and October 2022 were invited to participate in scheduled ophthalmological examinations in addition to routine dermatological examinations.

Before the start of dupilumab treatment, systemic immunosuppressive therapies were discontinued for at least 2 weeks. Patients were examined unblinded by a dermatologist and an ophthalmologist at three time points: prior to initiation of dupilumab treatment (baseline visit), after 28 weeks (early effect), and after at least 2 years of dupilumab treatment (last follow‐up visit, late effect). All patients were treated with dupilumab according to the label. At the last follow‐up visit, the dosage of dupilumab was tapered according to our standardized patient‐centered dosing regimen [15]. Patients who had tapered dupilumab to 300 mg every > 4 weeks prior to the last follow‐up visit were excluded.

The study was approved by the Medical Research Ethics Committee in Utrecht (METC 18/239), the Netherlands, as non‐interventional and was performed according to the Declaration of Helsinki. All patients provided written informed consent to participate in the BioDay registry and for the collection of tear fluid and conjunctival impression cytology (CIC) according to the Biobank protocol (17/884).

### 2.2 Data Collection

#### 2.2.1 Ophthalmological and Dermatological Examination

Ophthalmological examination was performed according to the Utrecht Ophthalmic Inflammatory and Allergic disease (UTOPIA) score, which assesses the severity of inflammation of the eyelids, conjunctiva (tarsal and bulbar), and limbus [11, 14]. OSD severity was categorized into no (score 0), mild (score 1–4), moderate (score 5–8), or severe (score ≥ 9) OSD, based on the eye with the highest UTOPIA score. DAOSD was defined as a ≥ 3‐point increase in UTOPIA score from baseline during dupilumab treatment, representing a pragmatic, non‐validated threshold for clinically relevant worsening of OSD.

AD severity was assessed by the Investigator's Global Assessment (IGA) and Eczema Area and Severity Index (EASI). Clinical baseline characteristics were extracted from the BioDay registry. Additionally, laboratory testing included thymus and activation‐regulated chemokine (TARC).

#### 2.2.2 Tear Fluid Collection

The tear break‐up time (TBUT) from both eyes was measured in seconds to examine tear film stability. Tear fluid production from both eyes was measured in millimetres (mm) by the Schirmer's test. After being air‐dried for at least one hour, the Schirmer's strips were stored at −80°C. Subsequently, the strips were eluted simultaneously to collect tear fluid as described previously [16]. Concentrations of dupilumab in tear fluid were measured with liquid chromatography tandem mass spectrometry according to a previously developed protocol at week 28 and the last follow‐up visit [16]. Tear fluid protein levels, including AD‐severity proteins and T‐helper (Th) 1‐, Th2‐, and Th17‐related proteins, were measured using Luminex technology at the Center of Translational Immunology (UMCU, the Netherlands) at baseline, week 28, and the last follow‐up visit (Supporting Information S1: Table S1) [17]. In addition, tear fluid protein levels were measured in 12 non‐atopic HCs with no medical history of any eye disease and no use of contact lenses.

#### 2.2.3 Conjunctival Impression Cytology

CIC was performed using an Eyeprim device to investigate the number of conjunctival GCs per mm^2^ according to the methods from previous studies [11, 18].In patients initiating dupilumab between October 2020 and February 2022, we additionally examined conjunctival GC function by investigating the percentage of cytokeratin 19 (CK19)‐CD45‐MUC5AC + cells and the median fluorescence intensity (MFI) of MUC5AC within MUC5AC + GCs, as described previously [11]. Only patients with available GC measurements at both baseline and the last follow‐up visit were included. CIC was also performed in 12 non‐atopic HCs.

### 2.3 Ophthalmic Treatment

Ophthalmic treatment was initiated in patients experiencing ocular symptoms or showing clinical signs of OSD. Standard ophthalmic treatment for mild OSD included lubricant eye drops (as needed), antihistamine eye drops, and tacrolimus skin ointment on the external eyelids. Depending on the severity of the OSD, additional ophthalmic treatments, such as non‐steroidal or steroidal anti‐inflammatory eye drops or ointments, could be used.

### 2.4 Statistical Analysis

The UTOPIA score, TBUT, and Schirmer's test were analyzed with a linear mixed model for the total cohort. A random intercept was included to correct for multiple measurements over time; time was included as a categorical variable. An additional residual covariance (i.e., GEE‐type) matrix was included to correct for the correlation between the left and right eye in patients [19]. Results were reported as estimated mean outcome values for each study visit and mean differences, all with 95% confidence intervals (CIs).

Unpaired comparisons were performed using the Mann‐Whitney *U* test (continuous variables) and the Chi‐square test or Fisher's exact test (categorical variables). Paired comparisons were performed using the Wilcoxon signed‐rank test (continuous variables) and the McNemar test (categorical variables). A *p* < 0.05 was considered statistically significant. Given the exploratory nature of the study, no formal correction for multiple testing was applied. The data were analyzed using IBM SPSS Statistics version 30.0.0 and SAS (v9.4, SAS Institute Inc.). GraphPad Prism version 10 was used to create the figures.

## 3 Results

### 3.1 Patient Characteristics

Forty‐four moderate‐to‐severe AD patients with a median EASI score of 14.0 (IQR 9.7–17.7) were included (Table 1). A history of self‐reported episodic acute allergic conjunctivitis was reported in 70.5% (*n* = 31/44) of patients. The median follow‐up time was 34.5 months (IQR 30.0–40.8). The flow chart of patient inclusion and exclusion is shown in Supporting Information S1: Figure S1.

**TABLE 1:** Patient characteristics at baseline.

Patient characteristics	Total cohort *N* = 44
Men, *n* (%)	24 (54.5)
Age at baseline (years), median (IQR)	39.7 (27.2–52.0)
Age of onset of AD^a^, *n* (%)	
Childhood	42 (95.5)
Adolescence	0 (0.0)
Adult	2 (4.5)
Atopic comorbidities, *n* (%)	
Allergic asthma	25 (56.8)
Self‐reported episodic acute allergic conjunctivitis	31 (70.5)
Allergic rhinitis	27 (61.4)
Food allergy	15 (34.1)
History of rosacea, *n (%)*	2 (4.5)
IGA score, median (IQR)	3.0 (3.0–3.0)
EASI score, median (IQR)^b^	14.0 (9.7–17.7)
AD eyelid involvement in the past year, *n* (%)	31 (70.5)
AD facial involvement in the past year, *n* (%)	38 (86.4)
TARC (pg/mL), median (IQR)	1207 (675–2038)
Medical history of non‐allergic ocular disease^c^, *n* (%)	11 (25.0)
Previously visited an ophthalmologist, *n* (%)	22 (50.0)
Previous use of ophthalmic medication, *n (%)*	25 (56.8)
Lubricants	9 (20.5)
Antihistamine eye drops	13 (29.5)
Tacrolimus skin ointment for the external eyelids	3 (6.8)
Non‐steroidal eye drops or ointment	2 (4.5)
Steroidal eye drops or ointment	2 (4.5)
Other/not specified	10 (22.7)

Abbreviations: AD, atopic dermatitis; EASI, Eczema Area and Severity Index; IGA, Investigator Global Assessment; IQR, interquartile range; TARC, Thymus‐ and Activation‐Regulated Chemokine.

^a^ Childhood, < 12 years; adolescence, 12–17 years; adult, ≥ 18 years.

^b^ Mild, EASI 0.1–5.9; moderate, EASI 6.0–22.9; severe, EASI 23.0–72.0.

^c^ Keratoconus (*n* = 1); blepharitis (*n* = 1); cataract (*n* = 1); herpetic keratitis (*n* = 3); amblyopia (*n* = 1); corpus alienum (*n* = 1); keratitis (*n* = 1); eye trauma (*n* = 1); nasolacrimal duct obstruction (*n* = 1).

### 3.2 Clinical Ophthalmological Characteristics and DAOSD

#### 3.2.1 Early Effect of Dupilumab Treatment (up to 28 Weeks)

At baseline, OSD characteristics were observed in 90.9% (*n* = 40/44) of patients, and this prevalence remained stable at week 28 (Table 2). The mean UTOPIA score showed a non‐significant reduction at week 28 compared to baseline of 0.45 (95% CI: −0.18 to 1.08, *p* = 0.159) (Supporting Information S1: Figure S2, Table S2). The number of patients reporting ocular symptoms was comparable at baseline (59.1%, *n* = 26/44) and week 28 (47.7%, *n* = 21/44). The use of ophthalmic treatment increased from 9.1% (*n* = 4/44) at baseline to 54.5% (*n* = 24/44) at week 28 (Supporting Information S1: Table S3). At week 28, the most commonly used ophthalmic treatments were antihistamine eye drops and tacrolimus skin ointment for the external eyelids (both 31.8%, *n* = 14/44), followed by anti‐inflammatory ophthalmic medication (18.2%, *n* = 8/44). The mean TBUT decreased non‐significantly at week 28 compared to baseline with 0.75 seconds (95% CI: −0.08 to 1.57, *p* = 0.075) (Figure 1, Supporting Information S1: Table S2). The mean tear fluid production increased significantly at week 28 compared to baseline with 2.52 mm (95% CI: 0.01–5.03, *p* = 0.049).

**TABLE 2:** Ophthalmological characteristics at baseline, week 28, and the last follow‐up visit.

Ophthalmological characteristics	Baseline *N* = 44	Week 28 *N* = 44	Last follow‐up *N* = 44
Presence of ocular symptoms, *n* (%)	26 (59.1)	21 (47.7)	26 (59.1)
Redness	8 (18.2)	10 (22.7)	3 (6.8)
Tearing	11 (25.0)	13 (29.5)	10 (22.7)
Pruritus	21 (47.7)	15 (34.1)	16 (36.4)
Pain	5 (11.4)	1 (2.3)	0 (0.0)
Photophobia	2 (4.5)	4 (9.1)	0 (0.0)
Burning	7 (15.9)	6 (13.6)	8 (18.2)
OSD severity, *n* (%)			
No OSD	4 (9.1)	4 (9.1)	4 (9.1)
Mild OSD	27 (61.4)	29 (65.9)	25 (56.8)
Moderate OSD	10 (22.7)	10 (22.7)	15 (34.1)
Severe OSD	3 (6.8)	1 (2.3)	0 (0.0)
Presence of OSD characteristics, *n* (%)			
Blepharitis	30 (68.2)	26 (59.1)	23 (52.3)
Meibomian gland dysfunction	26 (59.1)	23 (52.3)	34 (77.3)
Tarsal conjunctivitis	38 (86.4)	40 (90.9)	39 (88.6)
Bulbar conjunctivitis	23 (52.3)	19 (43.2)	17 (38.6)
Limbitis	2 (4.5)	3 (6.8)	3 (6.8)
Limbal vascularization	23 (52.3)	18 (40.9)	20 (45.5)
Punctate corneal lesions	11 (25.0)	11 (25.0)	10 (22.7)
Hurricane fluorescein staining	0 (0.0)	0 (0.0)	1 (2.3)
Goblet cells per mm^2^, median (IQR)	400 (220–660)	337 (124–680)	145 (42–281)
% CK19‐CD45‐MUC5AC + cells, median (IQR)	15.1 (9.1–31.9)	17.1 (9.7–28.5)	10.0 (2.1–45.4)
Missing, *n* (%)	16 (36.4)	19 (43.2)	16 (36.4)
Median fluorescence intensity	2659 (1481–3374)	1499 (1205–2110)	1524 (1224–2001)
Missing, *n* (%)	16 (36.4)	19 (43.2)	16 (36.4)

*Note:* Clinical characteristics of the most severe eye were reported.

Abbreviations: CK19, cytokeratin 19; IQR, interquartile range; MUC5AC, Mucin 5AC; OD, oculus dexter; OS, oculus sinister; OSD, ocular surface disease; TBUT, tear break‐up time; UTOPIA, Utrecht Ophthalmic Inflammatory and Allergic disease.

**FIGURE 1:**
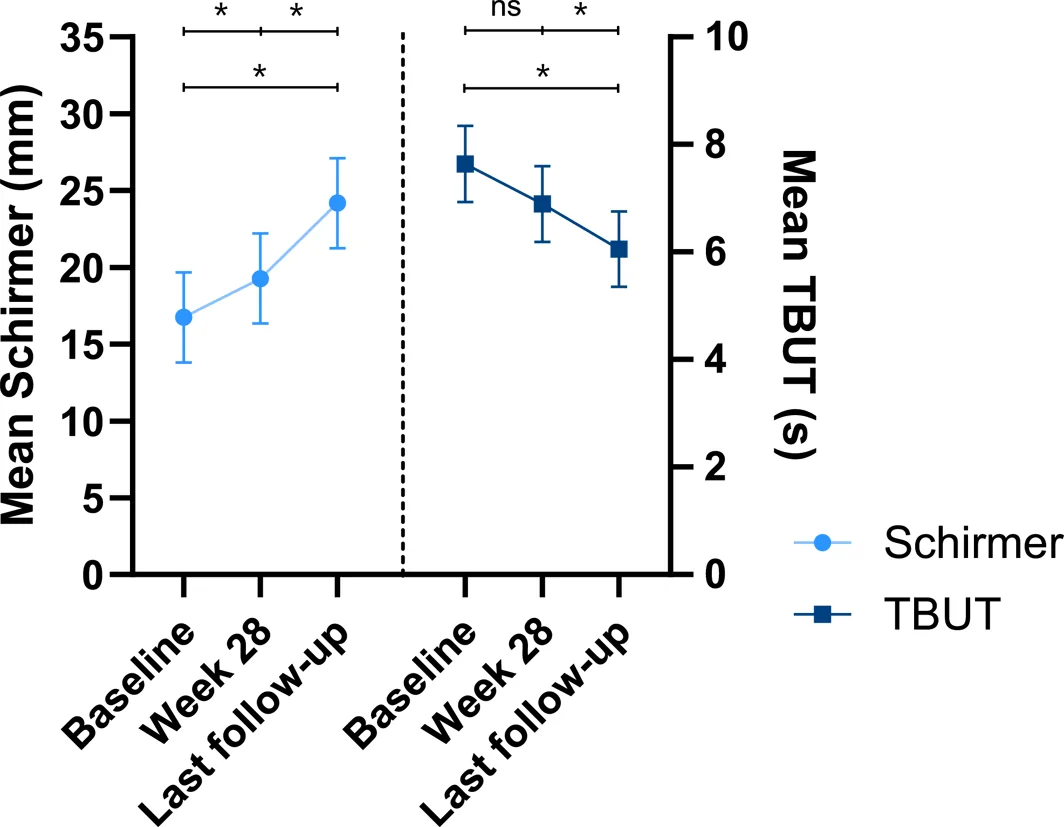
Estimated mean values of the Schirmer's test and tear break‐up time with 95% CI at baseline, week 28, and the last follow‐up visit. *p*‐values of < 0.05 were considered statistically significant. *Indicates statistical significance. CI, confidence interval; OS, oculus sinister; TBUT, tear break‐up time.

Between baseline and week 28, 13.6% (*n* = 6/44) of patients experienced worsening of OSD compatible with DAOSD (defined as a ≥ 3‐point increase in UTOPIA score from baseline), with a median time to onset of 8 weeks (IQR 4–13). All patients with DAOSD reported ocular symptoms; one of these patients was using ophthalmic treatment (i.e., antihistamine eye drops) at the time of DAOSD diagnosis.

#### 3.2.2 Late Effect of Dupilumab Treatment (After ≥ 2 Years)

At the last follow‐up visit, OSD characteristics were observed in 90.9% (*n* = 40/44) of patients (Table 2). The mean UTOPIA score showed a non‐significant reduction at the last follow‐up compared to baseline of 0.34 (95% CI: −0.29 to 0.97, *p* = 0.290) (Supporting Information S1: Figure S2, Table S2). The number of patients reporting ocular symptoms remained similar to baseline. At the last follow‐up visit, 38.6% (*n* = 17/44) of patients were using ophthalmic treatment, most frequently antihistamine eye drops and tacrolimus skin ointment for the external eyelids (both 18.2%, *n* = 8/44). Supporting Information S1: Table S4 presents ophthalmological outcomes stratified by ophthalmic medication use during follow‐up. The mean TBUT significantly decreased at the last follow‐up compared to baseline with 1.59 seconds (95% CI: 0.76–2.41, *p* < 0.001) (Figure 1, Supporting Information S1: Table S2). The mean tear fluid production significantly increased at the last follow‐up compared to baseline with 7.44 mm (95% CI: 4.92–9.95, *p* < 0.001).

From week 28 up to ≥ 2 years of follow‐up, 11.3% (*n* = 5/44) of patients had persistent (*n* = 1) or new‐onset (*n* = 4) DAOSD; all of these patients reported ocular symptoms. Among these DAOSD cases, 60.0% (*n* = 3/5) were using ophthalmic treatment, including antihistamine eye drops (*n* = 3) and oxytetracycline and hydrocortisone (*n* = 1). In patients with new‐onset DAOSD (9.1%, *n* = 4/44), the median time to onset was 117 weeks (IQR 51–171).

Overall, 22.7% (*n* = 10/44) of patients developed a ≥ 3‐point increase in UTOPIA score from baseline compatible with DAOSD during dupilumab treatment up to ≥ 2 years, with a median time to onset of 14 weeks (IQR 6–108). At diagnosis, the median UTOPIA score was 7.0 (IQR 4.8–9.3) (Supporting Information S1: Table S5). In all cases, ophthalmic treatment was initiated or intensified at DAOSD onset. Most patients (80.0%, *n* = 8/10) required non‐steroidal and/or steroidal anti‐inflammatory ophthalmic treatment (i.e., oxytetracycline/hydrocortisone eye ointment, dexamethasone eye drops, and ciclosporin eye drops) in addition to standard ophthalmic treatment, including lubricant eye drops (as needed), antihistamine eye drops, and tacrolimus skin ointment for the external eyelids. OSD severity improved afterward, with several patients being able to taper or discontinue anti‐inflammatory treatment. However, one patient still had persistent DAOSD at last follow‐up despite ongoing steroidal anti‐inflammatory treatment. For one patient, no follow‐up data were available.

### 3.3 Dupilumab Dosing Interval at the Last Follow‐Up Visit

At the last follow‐up visit, 53.2% (*n* = 23/44) of patients had tapered dupilumab Q2W to either every three (Q3W; 31.8%, *n* = 14/44) or 4 weeks (Q4W; 20.5%, *n* = 9/44) due to controlled AD (Supporting Information S1: Table S6). No dose adjustments were required specifically due to DAOSD. However, ocular symptoms contributed to the decision to taper dupilumab in 4.5% (*n* = 2/44) of patients. The median UTOPIA score was comparable between the Q2W and Q3W/Q4W group. The number of conjunctival GCs was higher in the Q3W/Q4W group than the Q2W group (185 [IQR 64–334] vs. 69 [IQR 31–235], *p* = 0.148, respectively), although not statistically significant.

### 3.4 Goblet Cell Numbers and MUC5AC Production

#### 3.4.1 Early Effect of Dupilumab Treatment (up to 28 Weeks)

At baseline, patients with moderate‐to‐severe AD had lower conjunctival GC numbers than HCs (400 [IQR 220–660] vs. 571 [IQR 395–863], *p* = 0.072, respectively), although this difference was not statistically significant (Figure 2A). No significant change in GC numbers was observed between baseline and week 28 (400 [220–660] vs. 337 [124–680], respectively). Similarly, the percentage of CK19‐CD45‐MUC5AC + cells remained stable over this period (Figure 2B). In contrast, the MFI of MUC5AC within CK19‐CD45‐MUC5AC + cells significantly decreased at week 28 compared to baseline (Figure 2C). These findings indicate an early decline in GC secretory function without a concomitant reduction in GC numbers during the first 28 weeks of dupilumab treatment.

**FIGURE 2:**
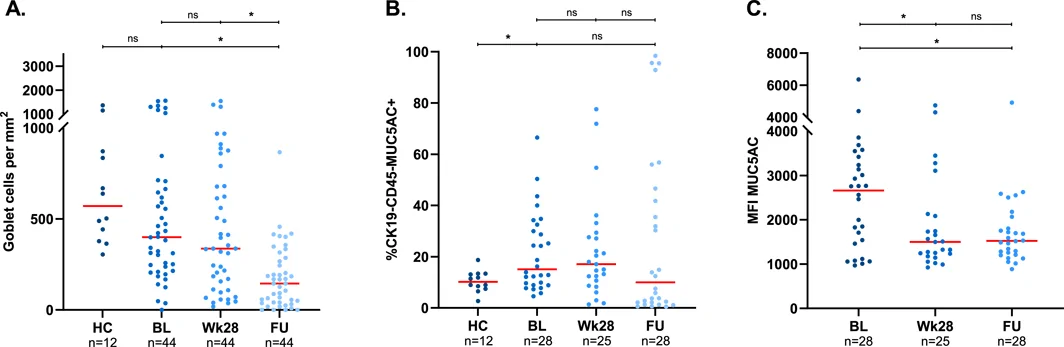
Conjunctival goblet cells at baseline, week 28, and the last follow‐up visit. *p*‐values of < 0.05 were considered statistically significant. *Indicates statistical significance. (A) Number of conjunctival goblet cells per square millimetre (mm^2^) at each time point. (B) Percentage of cytokeratin 19 (CK19)‐CD45‐Mucin 5AC (MUC5AC) + cells at each time point. (C) Median fluorescence intensity (MFI) of MUC5AC in CK19‐CD45‐MUC5AC + cells at each time point. CK, cytokeratin 19; HC, healthy controls; MFI, median fluorescence intensity; MUC5AC, Mucin 5AC; ns, not significant.

#### 3.4.2 Late Effect of Dupilumab Treatment (up to ≥ 2 Years)

At the last follow‐up visit, conjunctival GC numbers significantly decreased compared to both baseline and week 28 (145 [IQR 42–281] at last follow‐up, 400 [IQR 220–660] at baseline, and 337 [IQR 124–680] at week 28, *p* < 0.001) (Figure 2A). The percentage of CK19‐CD45‐MUC5AC + cells showed a non‐significant decrease at the last follow‐up compared to baseline and week 28 (Figure 2B). The MFI of MUC5AC within CK19‐CD45‐MUC5AC + cells remained significantly lower at the last follow‐up than at baseline, but did not further decrease compared to week 28 (Figure 2C). These findings demonstrate that long‐term dupilumab treatment is associated with a reduction in GC numbers, while the earlier decline in MUC5AC production persists without further functional deterioration.

### 3.5 Tear Fluid Dupilumab Levels

Tear fluid dupilumab levels were measured in 81.8% (*n* = 36/44) of patients. Overall, dupilumab tear fluid levels significantly decreased over time (0.20 mg/L [0.06–0.52] at week 28 vs. 0.05 mg/L [0.02–0.15] at the last follow‐up, *p* < 0.001) (Figure 3A). At the last follow‐up visit, dupilumab levels did not differ between dosing intervals (Q2W vs. Q3W/Q4W) (Figure 3B). No significant correlation was observed between dupilumab tear fluid levels and the time since the last dupilumab injection at either week 28 or the last follow‐up (Spearman's *ρ* = −0.085, *p* = 0.628 and *ρ* = −0.010, *p* = 0.955, respectively).

**FIGURE 3:**
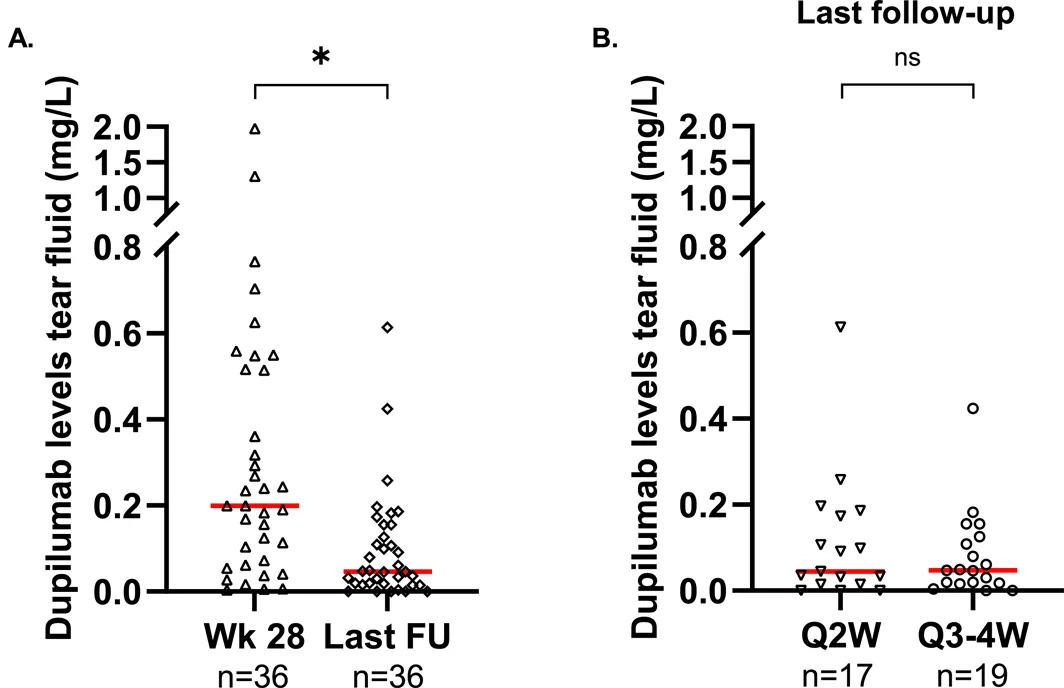
Dupilumab levels measured in tear fluid. Medians are indicated by red lines. *p*‐values of < 0.05 were considered statistically significant. *Indicates statistical significance. (A) Dupilumab levels in tear fluid at week 28 and the last follow‐up visit. (B) Dupilumab levels in tear fluid at the last follow‐up visit per dupilumab interval group. FU, follow‐up; ns, not significant; Q2W, every 2 weeks; Q3‐4W, every three to 4 weeks.

### 3.6 Tear Fluid Proteins

Tear fluid protein levels were measured in 79.5% (*n* = 35/44) of patients at baseline, 56.8% (*n* = 25/44) of patients at week 28%, and 79.5% (*n* = 35/44) of patients at the last follow‐up visit (Figure 4). At baseline, AD patients showed significantly higher tear fluid levels of periostin, TARC, and pulmonary and activation‐regulated chemokine (PARC) compared to HCs. During dupilumab treatment, levels of these AD‐severity proteins significantly decreased to levels comparable with HCs.

**FIGURE 4:**
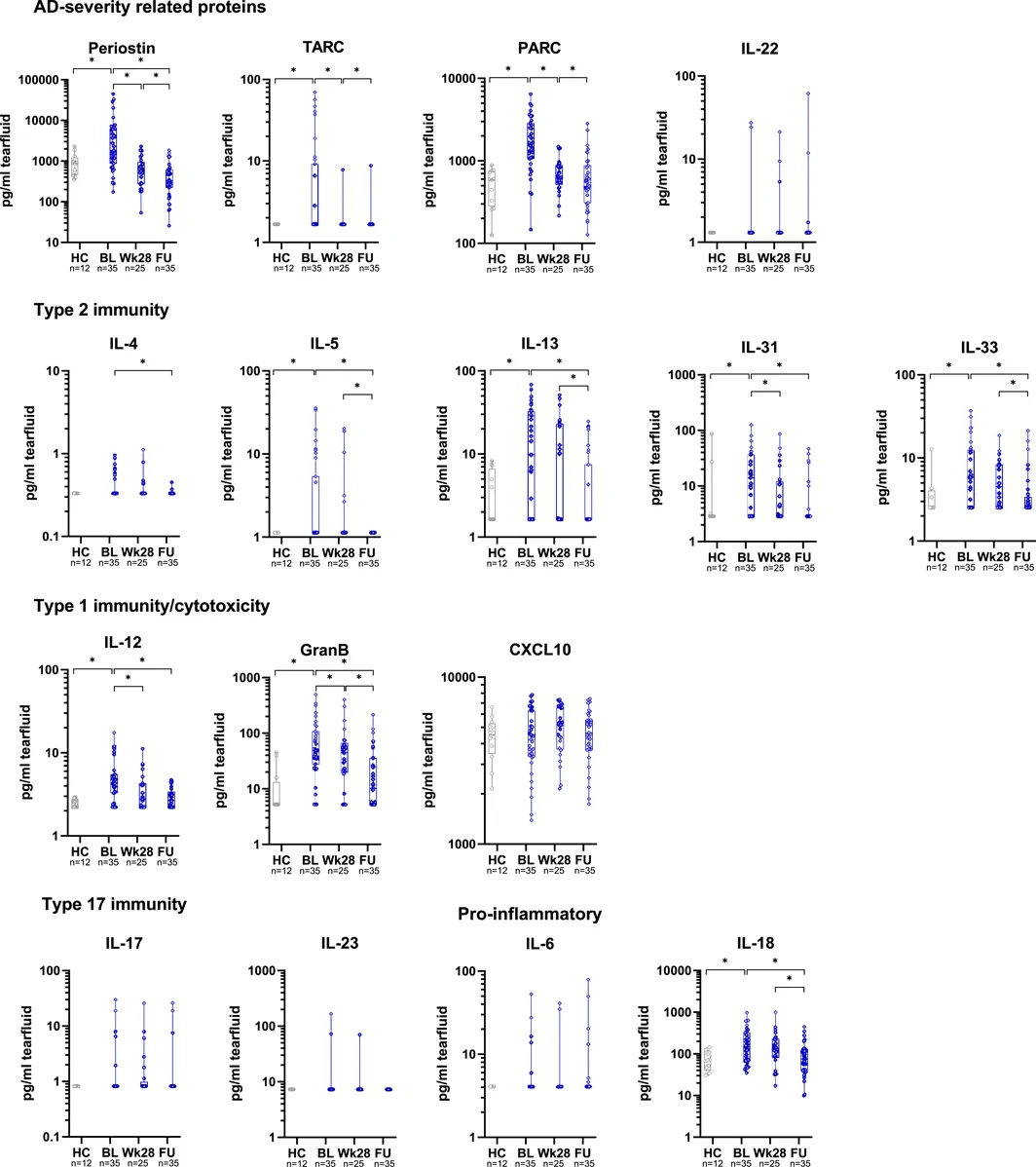
Tear fluid protein levels measured at baseline, week 28, and the last follow‐up visit. Boxplots with medians and interquartile ranges. Whisker plots display the minimum and maximum. *p*‐values of < 0.05 were considered statistically significant. *Indicates statistical significance. AD, atopic dermatitis; CXCL10, C‐X‐C motif chemokine ligand 10; FU, follow‐up; GranB, granzyme B; HC, healthy control; IL, interleukin; PARC, pulmonary and activation‐regulated chemokine; TARC, thymus and activation‐regulated chemokine; Th, T helper cell.

Tear fluid concentrations of the Th2‐related proteins IL‐5, IL‐13, IL‐31, and IL‐33 were significantly higher in AD patients than in HCs at baseline, and showed a significant and sustained decrease during dupilumab treatment. Similarly, the Th1‐related proteins IL‐12, IL‐18, and Granzyme B in tear fluid were significantly higher in AD patients than in HCs at baseline, and significantly decreased over time. No significant differences were observed for other Th1/Th17‐related proteins, including IL‐6, C‐X‐C motif chemokine ligand 10, IL‐17, and IL‐23.

Together, these results demonstrate that dupilumab treatment normalizes a broad spectrum of AD‐associated inflammatory mediators in tear fluid, particularly AD‐ and Th2‐related proteins, while leaving other Th1/Th17 pathways largely unaffected.

## 4 Discussion

This prospective cohort study shows that OSD remained common (90.9%, *n* = 40/44) and a subset of patients with moderate‐to‐severe AD experienced worsening of OSD compatible with DAOSD (22.7%, *n* = 10/44) during at least 2 years of dupilumab treatment. During follow‐up, dupilumab tear fluid levels and AD‐, Th2‐, and some Th1‐related tear fluid proteins significantly decreased. Although MUC5AC production by GCs decreased at week 28 and then remained stable, GC numbers significantly decreased at long‐term follow‐up, indicating an overall reduction in total MUC5AC production in dupilumab‐treated AD patients.

DAOSD is the most frequently reported side effect in dupilumab‐treated AD patients [4, 20]. However, long‐term ocular side effects of dupilumab have not yet been prospectively investigated through standardized ophthalmological examinations. Previous literature reported that most patients developed DAOSD within the first 26 weeks of treatment, but DAOSD can persist in 23% and 20.7% of DAOSD cases up to weeks 52 and 76, respectively [4, 5, 9, 13, 21]. In our study, 13.6% of patients (*n* = 6/44) developed a ≥ 3‐point increase in UTOPIA score from baseline compatible with DAOSD up to week 28, and 9.1% (*n* = 4/44) after week 28. Only one patient had persistent DAOSD at long‐term follow‐up. These findings indicate that DAOSD can persist or develop during long‐term dupilumab treatment, highlighting the ongoing need for clinicians to monitor ocular side effects in AD patients.

OSD is common in AD patients even before dupilumab initiation [22, 23, 24]. We identified OSD in 90.9% of patients at all time points. However, only half of the patients reported ocular symptoms, which highlights that patient‐reported diagnosis is not reliable in diagnosing OSD in AD patients [6, 25, 26]. After at least 2 years of dupilumab treatment, patients showed increased tear fluid production, decreased tear film stability, and more meibomian gland dysfunction compared to baseline and/or week 28. These findings suggest worsening of OSD manifestations over time [25]. Nevertheless, median UTOPIA scores slightly improved at week 28 and the last follow‐up visit compared to baseline, probably due to the increased use of ophthalmic medication and increased tear fluid production. The stable frequency of patient‐reported ocular symptoms may reflect habituation to ocular discomfort and the effect of ophthalmic medication.

Currently, the exact pathomechanism of DAOSD remains unknown. However, dupilumab affects conjunctival GC proliferation and mucin secretion by blocking IL‐4 and IL‐13, which are both expressed on the conjunctival epithelium [6, 27, 28, 29]. We found lower GC numbers in moderate‐to‐severe AD patients than in HCs. Unlike our previous study, this difference did not reach statistical significance [6, 11, 30]. Notably, the median GC count in HCs was comparable to that reported by Doughty (511 cells/mm^2^) [6, 11, 30]. Previous studies showed that conjunctival GCs decreased after 16 weeks of dupilumab treatment, while other studies reported low but stable GC numbers with a reduced GC function [6, 27, 29]. In our cohort, MUC5AC production in CK19‐CD45‐MUC5AC + cells significantly decreased between baseline and week 28, while GC numbers remained stable. Between week 28 and the last follow‐up, MUC5AC production per cell stabilized, however, GC numbers declined significantly. Together, this suggests that dupilumab induced early impairment of GC secretory function, whereas prolonged IL‐4/IL‐13 blockade may contribute to progressive GC depletion during long‐term treatment. Overall, these findings indicate a sustained reduction in conjunctival mucin‐producing capacity at long‐term follow‐up, although confirmation in larger studies is needed.

In addition to an overall reduction in MUC5AC at long‐term, patients showed an increased prevalence of meibomian gland dysfunction and decreased tear film stability (TBUT) at the last follow‐up visit. Although the reduction in TBUT was statistically significant, a decrease of 1.59 seconds may not necessarily translate into clinically meaningful symptoms. The increased tear fluid production (Schirmer's test) may reflect a compensatory or reflex response to compensate for the overall decrease in MUC5AC and lipid production by the meibomian glands. Despite this potential compensatory mechanism, the strong reduction in MUC5AC raises concerns about the potential long‐term effects of dupilumab on the ocular surface, given the important protective role of MUC5AC. However, further research is needed to clarify the clinical implications of decreased GC numbers and MUC5AC production.

Our previous study showed that extending dupilumab dosing intervals to every 3–5 weeks due to conjunctivitis improved DAOSD in most patients [14]. Although reduced systemic exposure could theoretically affect the occurrence or severity of ocular adverse events, we observed no significant differences in OSD severity between the Q2W and Q3W/Q4W groups in the present study. This discrepancy might be explained by differences in the indication for interval prolongation: in the present study, the interval was extended due to well‐controlled AD and was not based on DAOSD. However, given the limited sample sizes of both subgroups, these analyses were likely underpowered to detect small differences. Therefore, future studies with larger sample sizes are needed to further investigate the effect of interval prolongation on ocular outcomes.

Tear fluid analysis showed higher levels of AD‐, Th2‐, and some Th1‐related proteins in AD patients at baseline compared to HCs. These elevated protein levels suggest pre‐existing AD‐related ocular inflammation, which is consistent with the high baseline prevalence of OSD (90.9%, *n* = 40/44). During dupilumab treatment, Th2‐related proteins showed a sustained and significant reduction, which may reflect dupilumab's local mode of action. AD‐severity protein levels (i.e., periostin, TARC, and PARC) also significantly decreased during treatment, which may indicate a potential beneficial effect on AD‐related OSD and a partial restoration of the blood‐tear barrier due to reduced ocular inflammation. We hypothesize that this potential barrier restoration could contribute to the significantly lower dupilumab tear fluid levels observed at the last follow‐up compared to week 28. Overall, our results suggest that dupilumab suppresses AD‐ and Th2‐related tear fluid proteins.

The increased use of ophthalmic medication in our cohort raises questions regarding its effect on ophthalmological outcomes, including ocular symptoms, UTOPIA scores, tear film parameters, tear fluid protein levels, and conjunctival GCs. The initiation of ophthalmic treatment was primarily guided by OSD severity and therefore complicates the interpretation of ophthalmological outcomes. Anti‐inflammatory treatment, including ocular corticosteroids and tacrolimus skin ointment for the external eyelids, has been shown to suppress conjunctival inflammatory markers and may increase GC numbers and possibly GC function [31, 32, 33]. In contrast, lubricants improve tear film stability but do not seem to significantly affect GCs [34, 35, 36]. Exploratory analyses comparing patients with and without ophthalmic medication during follow‐up did not demonstrate a consistent pattern suggesting that ophthalmic treatment explained the observed findings. However, these analyses should be interpreted with caution because of the limited sample size and the potential for confounding by indication. Consequently, a potential influence of ophthalmic treatment on ophthalmological outcomes cannot be completely excluded and should be considered when interpreting these findings.

To the best of our knowledge, this is the first prospective cohort study investigating ophthalmological outcomes in dupilumab‐treated AD patients over an extended 2‐year follow‐up period. A strength of this study is the standardized ophthalmological examinations (i.e., UTOPIA score) before and during dupilumab treatment, which makes the outcome and comparison over time reliable. For the UTOPIA score, TBUT, and Schirmer's test, a mixed model was applied to evaluate changes over time in both eyes simultaneously, thus optimizing statistical power. Limitations include the relatively small sample sizes, which may affect the generalizability of the findings. In addition, the lack of a formal sample size calculation may have limited statistical power. Furthermore, our definition of DAOSD (≥ 3‐point increase in UTOPIA score from baseline) represents a pragmatic, non‐validated threshold to identify clinically relevant worsening of OSD. However, it does not prove a causal relationship with dupilumab and could also represent progression or fluctuation of pre‐existing AD‐related OSD. Nevertheless, we believe that the use of a clinical scoring system provides more objective results than relying solely on patient‐reported outcomes [26]. Finally, only patients still receiving dupilumab at the last follow‐up visit were included in the study, which may have resulted in an underestimation of (DA)OSD and limits generalizability. However, data from our BioDay registry showed a low discontinuation rate due to DAOSD (3.0%) over 5 years, suggesting this potential underestimation is limited [37].

## 5 Conclusion

In conclusion, in this selected cohort of patients with moderate‐to‐severe AD and a high baseline OSD burden, OSD remained common during long‐term dupilumab treatment. In addition, a subset of patients developed worsening of OSD compatible with DAOSD, including cases with a delayed onset, highlighting the need for continued awareness among clinicians of potential long‐term ocular side effects. Exploratory findings from impression cytology and tear‐fluid analyses suggest an overall decrease in MUC5AC at long‐term follow‐up, potentially compromising tear film stability and conjunctival barrier function. In contrast, the decrease in AD‐related inflammatory protein levels in tear fluid may indicate a potential beneficial effect on AD‐related OSD. Future studies with larger cohorts are needed to validate these findings and to determine whether these findings translate into clinically relevant ophthalmological consequences during long‐term dupilumab treatment.

## Author Contributions

**Nienke Veldhuis:** conceptualization, data curation, formal analysis, investigation, methodology, project administration, software, visualization, writing – original draft, writing – review and editing. **Roselie E. Achten:** conceptualization, investigation, methodology, supervision, visualization, writing – review and editing. **Chantal M. van Luijk:** investigation, writing – review and editing. **Marlot M. van der Wal:** investigation, writing – review and editing. **Constance F. den Hartog Jager:** investigation, writing – review and editing. **Nicolaas P. A. Zuithoff:** formal analysis, methodology, software, visualization, writing – review and editing. **Mohsin El Amrani:** investigation, resources, writing – review and editing. **Edward F. Knol:** investigation, resources, writing – review and editing. **Femke van Wijk:** conceptualization, methodology, resources, supervision, visualization, writing – review and editing. **Marlies de Graaf:** conceptualization, funding acquisition, methodology, resources, supervision, visualization, writing – review and editing. **Inge M. Haeck:** conceptualization, methodology, resources, supervision, visualization, writing – review and editing. **Mor M. Dickman:** conceptualization, methodology, resources, supervision, visualization, writing – review and editing. **Marjolein S. de Bruin‐Weller:** conceptualization, funding acquisition, methodology, resources, supervision, visualization, writing – review and editing.

## Funding

Patients included in this manuscript participated in the BioDay registry sponsored by Sanofi/Regeneron, Eli Lilly and Company, Almirall, LEO Pharma, and Pfizer. The sponsors were not involved in the collection, analysis, interpretation, and publication of the data.

## Ethics Statement

All included patients provided written informed consent to participate in the BioDay registry (Medical Research Ethics Committee, University Medical Center Utrecht; 18/239). In addition, all included patients provided written informed for the collection of blood, tear fluid, and conjunctival impression cytology (CIC) according to the Biobank protocol (17/884).

## Conflicts of Interest

N. Veldhuis has been a speaker for Sanofi. N. P. A. Zuithoff has been involved in studies financed by Eli Lilly. Edward F. Knol has previously served as a speaker and consultant for Sanofi, Thermo Fisher Scientific, Astra Zeneca, Zoetis and GSK. F. van Wijk has been a speaker and/or consultant for Janssen, Johnson & Johnson, and Takeda and has received grants from Regeneron Pharmaceuticals, Leo Pharma, Sanofi, BMS, Galapagos, and Takeda. M. de Graaf has been a consultant, advisory board member, and/or speaker for AbbVie, ALK, Almirall, Eli Lilly, Galderma, Janssen, Leo Pharma, Novartis, Pfizer, Regeneron Pharmaceuticals, and Sanofi. I. M. Haeck has been a consultant, advisory board member, and/or speaker for Sanofi and Regeneron Pharmaceuticals, LEO Pharma, AbbVie, Janssen, and Eli Lilly. M. M. Dickman has has nothing to disclose. M. S. de Bruin‐Weller has been a consultant, advisory board member, and/or speaker for AbbVie, Almirall, Aslan, Eli Lilly, Galderma, Leo Pharma, Novartis, Takeda, Pfizer, Regeneron Pharmaceuticals, and Sanofi. R. E. Achten, C. M. van Luijk, M. M. van der Wal, C. F. den Hartog Jager, and M. El Amrani have nothing to disclose.

## Supporting information


Supporting Information S1

## Data Availability

The data that support the findings of this study are available from the corresponding author upon reasonable request.
