# Supplementary material for: Long‐Term Ocular Outcomes of Dupilumab in Atopic Dermatitis: Results From the BioDay Registry
Source: Clin Transl Allergy. 2026 Sep 29;16(10):e70208. doi: 10.1002/clt2.70208 (PMC13621575; doi:10.1002/clt2.70208)
Supplement: Supplementary file 1 [file CLT2-16-e70208-s001.docx]

**Table S1. Panel of measured proteins in tear fluid**

| AD-severity related | Periostin/OSF2  TARC/CCL17  PARC/CCL18  IL-22 |
| --- | --- |
| Type 2 immunity | IL-4  IL-5  IL-13  IL-31  IL-33 |
| Type 1 immunity/cytotoxicity | IL-12  GranB IP10/CXCL10 |
| Th17 immunity | IL-17  IL-23 |
| Pro-inflammatory | IL-6  IL-18 |

Abbreviations: AD, atopic dermatitis; CXCL10, C-X-C motif chemokine ligand 10; GranB, granzyme B; IL, interleukin; PARC, pulmonary and activation-regulated chemokine; TARC, thymus and activation-regulated chemokine; Th, T helper cel

**Table S2. Estimated mean values of the UTOPIA score, Schirmer’s test, and tear break-up time at baseline, week 28, and the last follow-up visit.**

| Ophthalmological characteristics | Estimated mean (95% CI) |
| --- | --- |
| UTOPIA score, ODS |  |
| Baseline | 3.61 (2.99-4.23) |
| Week 28 | 3.16 (2.53-3.78) |
| Last follow-up | 3.27 (2.65-3.89) |
| Schirmer’s test, ODS, mm |  |
| Baseline | 16.75 (13.82-19.68) |
| Week 28 | 19.27 (16.34-22.20) |
| Last follow-up | 24.18 (21.26-27.11) |
| TBUT, ODS, seconds |  |
| Baseline | 7.64 (6.93-8.35) |
| Week 28 | 6.89 (6.19-7.60) |
| Last follow-up | 6.05 (5.35-6.76) |

Abbreviations: CI, confidence interval; ODS, oculus dexter sinister; TBUT, tear break-up time; UTOPIA, Utrecht Ophthalmic Inflammatory and Allergic disease

**Table S3. Number of patients treated with ophthalmic medication at baseline, week 28, and the last follow-up visit during dupilumab treatment.**

| Ophthalmic treatment per medication group, *n (%)* | Baseline  N=44 | Week 28  N=44 | Last follow-up  N=44 |
| --- | --- | --- | --- |
| No medication | 40 (90.9) | 20 (45.5) | 27 (61.4) |
| Lubricants | 1 (2.3) | 5 (11.4) | 6 (13.6) |
| Antihistamine eye drops | 1 (2.3) | 14 (31.8) | 8 (18.2) |
| Tacrolimus skin ointment for the external eyelids | 0 (0.0) | 14 (31.8) | 8 (18.2) |
| Anti-inflammatory eye drops or ointment (non-steroidal) | 0 (0.0) | 1 (2.3) | 0 (0.0) |
| Anti-inflammatory eye drops or ointment (steroidal) | 1 (2.3) | 7 (15.9) | 3 (6.8) |
| Other | 1 (2.3)^†^ | 1 (2.3)^†^ | 0 (0.0) |

^†^ One patient treated with valaciclovir due to herpetic keratitis.

**Table S4. Ophthalmological characteristics stratified by ophthalmic medication use at baseline, week 28, and the last follow-up visit**

|  | Ophthalmic medication at baseline | | Ophthalmic medication at week 28 | | Ophthalmic medication at last follow-up | |
| --- | --- | --- | --- | --- | --- | --- |
|  | **No**  **N=40** | **Yes**  **N=4** | **No**  **N=20** | **Yes**  **N=24** | **No**  **N=27** | **Yes**  **N=17** |
| Presence of ocular symptoms, n (%) | 22 (55.0) | 4 (100.0) | 5 (25.0) | 16 (66.7) | 14 (51.9) | 12 (70.6) |
| OSD severity, n (%) |  |  |  |  |  |  |
| No OSD | 4 (10.0) | 0 (0.0) | 3 (15.0) | 1 (4.2) | 2 (7.4) | 2 (11.8) |
| Mild OSD | 24 (60.0) | 3 (75.0) | 13 (65.0) | 16 (66.7) | 18 (66.7) | 7 (41.2) |
| Moderate OSD | 10 (25.0) | 1 (25.0) | 3 (15.0) | 7 (29.2) | 7 (25.9) | 8 (47.1) |
| Severe OSD | 2 (5.0) | 0 (0.0) | 1 (5.0) | 0 (0.0) | 0 (0.0) | 0 (0.0) |
| UTOPIA score, median (IQR) | 4.0 (2.3-5.0) | 4.0 (2.5-7.8) | 3.0 (2.0-4.0) | 3.0 (2.0-5.0) | 3.0 (2.0-5.0) | 4.0 (2.0-6.0) |
| Schirmer OS, median (IQR) | 15.0 (10.0-24.0) | 6.5 (2.3-28.8) | 19.0 (11.0-32.0) | 16.5 (7.5-33.8) | 26.0 (13.0-35.0) | 30.0 (25.5-35.0) |
| Schirmer OD, median (IQR) | 18.0 (10.3-24.0) | 8.0 (2.5-28.5) | 19.5 (15.3-31.8) | 14.5 (6.3-31.3) | 23.0 (15.0-33.0) | 24.0 (17.0-35.0) |
| TBUT OS, median (IQR) | 9.0 (6.0-10.0) | 5.0 (3.3-9.0) | 7.0 (5.0-9.0) | 7.5 (5.0-10.0) | 5.0 (4.0-8.0) | 5.0 (4.0-8.0) |
| Missing, n (%) | 1 (2.5) | 0 (0.0) | 0 (0.0) | 0 (0.0) | 0 (0.0) | 0 (0.0) |
| TBUT OD, median (IQR) | 9.0 (5.0-10.0) | 6.0 (3.3-9.5) | 5.0 (5.0-8.5) | 7.0 (5.0-10.0) | 6.0 (5.0-8.0) | 5.0 (4.0-7.0) |
| Missing, n (%) | 1 (2.5) | 0 (0.0) | 0 (0.0) | 0 (0.0) | 0 (0.0) | 0 (0.0) |
| Goblet cells per mm^2^, median (IQR) | 400 (240-660) | 315 (77-1050) | 201 (68-351) | 497 (263-881) | 127 (32-286) | 168 (61-323) |
| %CK19-CD45-MUC5AC+ cells, median (IQR) | 12.8 (8.9-27.6) | 33.6 (27.0-38.7) | 27.6 (14.7-43.9) | 13.9 (8.4-22.0) | 5.8 (2.1-31.9) | 12.4 (2.5-56.4) |
| Missing, n (%) | 16 (40.0) | 0 (0.0) | 11 (55.0) | 8 (33.3) | 12 (44.4) | 4 (23.5) |
| Median fluorescence intensity | 2659 (1481-3210) | 2758 (1253-5686) | 1326 (1254-3278) | 1505 (1153-2001) | 1589 (1293-2067) | 1343 (1090-1991) |
| Missing, n (%) | 16 (40.0) | 0 (0.0) | 11 (55.0) | 8 (33.3) | 12 (44.4) | 4 (23.5) |

Clinical characteristics of the most severe eye were reported. Abbreviations: CK19, cytokeratin 19; IQR, interquartile range; OD, oculus dexter; OS, oculus sinister; OSD, ocular surface disease; TBUT, tear break-up time; UTOPIA, Utrecht Ophthalmic Inflammatory and Allergic disease.

**Table S5. Ophthalmological characteristics in AD patients who developed dupilumab-associated ocular surface disease during dupilumab treatment**

| Ophthalmological characteristics | Baseline  N=10 | Onset of DAOSD^†^  N=10 | *P*-value |
| --- | --- | --- | --- |
| Time until onset of DAOSD, weeks, median (IQR) | NA | 13.5 (5.5-108.3) | NA |
| Dupilumab dose interval, *n (%)* |  |  |  |
| Q2W | NA | 8 (80.0) | NA |
| Q3W | NA | 0 (0.0) | NA |
| Q4W | NA | 2 (20.0) | NA |
| Presence of ocular symptoms, *n (%)* | 8 (80.0) | 9 (90.0) | 1.000 |
| Redness | 2 (20.0) | 5 (50.0) | 0.250 |
| Tearing | 4 (40.0) | 6 (60.0) | 0.687 |
| Pruritus | 6 (60.0) | 8 (80.0) | 0.500 |
| Pain | 0 (0.0) | 5 (50.0) | 0.063 |
| Photophobia | 0 (0.0) | 2 (20.0) | 0.500 |
| Burning | 1 (10.0) | 3 (30.0) | 0.500 |
| UTOPIA score, median (IQR) | 2.0 (0.0-3.5) | 7.0 (4.8-9.3) | **0.005** |
| OSD severity, *n (%)* |  |  |  |
| No OSD | 3 (30.0) | 0 (0.0) | 0.250 |
| Mild OSD | 5 (50.0) | 2 (20.0) | 0.375 |
| Moderate OSD | 2 (20.0) | 5 (50.0) | 0.453 |
| Severe OSD | 0 (0.0) | 3 (30.0) | 0.250 |
| Presence of OSD characteristics, *n (%)* |  |  |  |
| Blepharitis | 5 (50.0) | 10 (100.0) | 0.063 |
| Meibomian gland dysfunction | 5 (50.0) | 8 (80.0) | 0.250 |
| Tarsal conjunctivitis | 5 (50.0) | 10 (100.0) | 0.063 |
| Bulbar conjunctivitis | 2 (20.0) | 9 (90.0) | **0.016** |
| Limbitis | 0 (0.0) | 0 (0) | NA |
| Limbal vascularization | 3 (30.0) | 8 (80.0) | 0.063 |
| Punctate corneal  lesions | 1 (10.0) | 4 (40.0) | 0.250 |
| Hurricane fluorescein staining | 0 (0.0) | 1 (10.0) | 1.000 |
| Concomitant ophthalmic treatment, *n (%)* | 1 (10.0) | 5 (50.0) | 1.000 |
| TBUT OD, seconds, median (IQR) | 5 (4-10) | 7 (5-10) | 0.586 |
| *Missing, n (%)* | 1 (10.0) | 0 (0.0) | NA |
| TBUT OS, seconds, median (IQR) | 8 (4-10) | 10 (3-10) | 0.893 |
| *Missing, n (%)* | 1 (10.0) | 0 (0.0) | NA |
| Schirmer’s test OD, millimetre, median (IQR) | 22 (12-25) | 25 (12-31) | 0.260 |
| Schirmer’s test OS, millimetre, median (IQR) | 24 (10-30) | 26 (12-33) | 0.262 |
| Goblet cells per mm^2^, median (IQR) | 276 (209-721) | 240 (64-407) | 0.059 |

*P*-values of <0.05 were considered as statistically significant. Clinical characteristics of the most severe eye were reported. ^†^ Increase in UTOPIA score of ≥ 3 points from baseline. Abbreviations: DAOSD, dupilumab-associated ocular surface disease; IQR, interquartile range; NA, not applicable; OD, oculus dexter; OS, oculus sinister; OSD, ocular surface disease; Q2W, every two weeks; Q3W, every three weeks; Q4W, every four weeks; TBUT, tear break-up time; UTOPIA, Utrecht Ophthalmic Inflammatory and Allergic disease.

**Table S6. Ophthalmological characteristics by dupilumab dose interval at the last follow-up visit**

| Ophthalmological characteristics | Dupilumab  Q2W  N=21 | Dupilumab  Q3W/Q4W  N=23 | *P*-value |
| --- | --- | --- | --- |
| Presence of ocular symptoms, *n (%)* | 11 (52.4) | 15 (65.2) | 0.387 |
| UTOPIA score, median (IQR) | 3.0 (2.0-5.0) | 4.0 (2.0-5.0) | 0.577 |
| OSD severity, *n (%)* |  |  |  |
| No OSD | 1 (4.8) | 3 (13.0) | 0.609^†^ |
| Mild OSD | 13 (61.9) | 12 (52.2) | 0.515 |
| Moderate OSD | 7 (33.3) | 8 (34.8) | 0.919 |
| Severe OSD | 0 (0.0) | 0 (0.0) | NA |
| TBUT OS, seconds, median (IQR) | 5 (4-7) | 6 (4-9) | 0.114 |
| TBUT OD, seconds, median (IQR) | 5 (4-7) | 7 (5-8) | 0.090 |
| Schirmer’s test OS, mm, median (IQR) | 26 (19-35) | 29 (13-35) | 0.866 |
| Schirmer’s test OD, mm, median (IQR) | 23 (19-33) | 26 (15-35) | 0.785 |
| Goblet cells per mm^2^, median (IQR) | 69 (31-235) | 185 (65-334) | 0.148 |
| % CK19-CD45-MUC5AC+ cells, median (IQR) | 12.4 (2.1-92.9) | 7.5 (1.8-31.1) | 0.413 |
| Median fluorescence intensity | 1721 (1293-2505) | 1467 (1173-1615) | 0.142 |

*P*-values of <0.05 were considered statistically significant. Clinical characteristics of the most severe eye were reported. ^†^ Fisher's exact test was used due to low expected counts. Abbreviations: CK19, cytokeratin 19; IQR, interquartile range; MUC5AC, Mucin 5AC; NA, not applicable; OD, oculus dexter; OS, oculus sinister; OSD, ocular surface disease; Q2W, every two weeks; Q3W, every three weeks; Q4W, every 4 weeks; TBUT, tear break-up time; UTOPIA, Utrecht Ophthalmic Inflammatory and Allergic disease.

**Figure S1. Flow chart of patient inclusion and reasons for exclusion.** Figure created with Biorender.

**Figure S2. Estimated mean values of the UTOPIA score with 95% CI at baseline, week 28, and the last follow-up visit.** *P*-values of <0.05 were considered statistically significant. Abbreviations: CI, confidence interval; ns, not significant.
